# CIRBP Regulates Pancreatic Cancer Cell Ferroptosis and Growth by Directly Binding to p53

**DOI:** 10.1155/2022/2527210

**Published:** 2022-08-25

**Authors:** Hongqiang Gao, Ran Xie, Rong Huang, Chonglin Wang, Yue Wang, Dongdong Wang, Kaimin Liu, Conghui Yang, Qingxiong Yang, Long Chen

**Affiliations:** ^1^Department of Hepatobiliary Surgery, The First People's Hospital of Kunming City & Ganmei Affiliated Hospital of Kunming Medical University, No. 504 Youth Road, Kunming 650000, China; ^2^Department of PET/CT Center, Yunnan Cancer Hospital, The Third Affiliated Hospital of Kunming Medical University, Yunnan, China; ^3^Department of Radiology, Kunming Children's Hospital, Yunnan, China

## Abstract

Pancreatic cancer is one of the most malignant gastrointestinal tumors, and it is of great significance to explore the molecular mechanism of its progression and find new biological therapeutic targets. CIRBP is a cold-induced protein that plays a key role in many physiological and pathological processes, but its role in pancreatic cancer is still unclear. The expression of CIRBP in pancreatic cancer tissues was slightly lower than that in normal tissues, and the high expression of CIRBP was beneficial to survival. At the same time, immunohistochemical detection showed that the expression level of CIRBP in the cytoplasm of cancer tissues was significantly lower than that of adjacent tissues; survival curve analysis showed that pancreatic cancer patients with high nuclear CIRBP expression had a longer overall survival period. RIP results showed that CIRBP antibody significantly enriched p53 RNA, indicating that it could directly bind to p53. Cold treatment of pancreatic cancer cells significantly induced the expression of CIRBP, DPP4, NOX1, and FTH1 and inhibited the expression of p53 and GPX4. Cold induction enhanced the accumulation of Fe^2+^ in cells, promoted the generation of ROS, and inhibited the expression of GSH-Px. Therefore, cold induction promotes the process of ferroptosis by inducing the expression of CIRBP and then regulating key factors such as p53 and GPX4. In addition, cold induction significantly inhibited the proliferation of pancreatic cancer cells and induced cell apoptosis, but after the addition of ferroptosis inhibitor, cell proliferation and apoptosis did not change significantly. Therefore, CIRBP acts as a tumor suppressor gene in pancreatic cancer and induces ferroptosis through the p53/GPX4 pathway to inhibit cell growth, which may be an important target for the diagnosis and treatment of pancreatic cancer.

## 1. Introduction

Pancreatic cancer (PC) is one of the most malignant tumors, which is highly invasive and characterized by high mortality and poor prognosis [1]. In recent years, its incidence is gradually increasing. It is estimated that by 2030, pancreatic cancer will become the second leading cause of cancer-related death after lung cancer [2]. Therefore, the current clinical work urgently needs new effective biomarkers to provide ideas for diagnosis or treatment.

Cold-inducible RNA-binding protein (CIRBP), as an RNA-binding protein that is abundantly expressed under cold stress, widely participates and plays an important role in various physiological processes of cells [3]. In recent years, studies have gradually found that CIRBP also plays an important role in the occurrence and development of tumors [3]. For example, Zeng et al. [4] found that in prostate cancer, CIRBP knockdown inhibited tumor cell proliferation and enhanced its chemotherapy sensitivity. CIRBP is highly expressed in lung cancer, breast cancer, and bladder cancer, and its expression level is negatively correlated with the prognosis of patients [5]. However, at the same time, some studies have shown that CIRBP is lowly expressed in nasopharyngeal carcinoma and colorectal cancer and can be used as a marker of tumor prognosis [6, 7]. Therefore, CIRBP may play different roles in different tumor progression, and its role in pancreatic cancer is still poorly studied.

Ferroptosis is an iron-dependent nonapoptotic cell death discovered in recent years. Its process is closely related to the accumulation of iron ions and lipid reactive oxygen species (ROS) in cells and is involved in the occurrence and development of many diseases [8]. At present, a large number of research results show that inducing tumor cell ferroptosis has important therapeutic significance [9], and the correlation between ferroptosis and pancreatic cancer is also increasing [10], which is expected to become a new therapeutic direction. For example, Song et al. [11] found that ruscogenin regulated transferrin to increase intracellular Fe^2+^ concentration and ROS levels in human pancreatic cancer cells, and this effect was inhibited by deferoxamine, suggesting that ruscogenin may play an antipancreatic cancer effect by inducing ferroptosis.

Studies have found that the function of CIRBP is related to p53 [12], and p53 also plays an important regulatory role in the progression of ferroptosis [13]. Therefore, CIRBP may play a role in ferroptosis through p53. In order to verify the above inferences, this study specifically explored the mechanism of CIRBP in pancreatic cancer through clinical detection and cell experiments.

## 2. Materials and Methods

### 2.1. Clinical Specimens and Bioinformatics Analysis

A total of 90 pancreatic cancer tissues and 90 paratumor normal pancreatic tissues were collected in this study. All pancreatic cancer patients did not receive any neoadjuvant therapy before tumor resection. Written informed consent was provided by all patients, and the study was approved by the Institute's Ethics Committee. The expression level of CIRBP was checked by using the online tool GEPIA2 (http://gepia2.cancer-pku.cn/#index).

### 2.2. Immunohistochemistry

Freshly cut pancreatic cancer tissue microarray (HPan-Ade180Sur-01; Outdo, Shanghai, China) sections were immunostained to measure the expression of CIRBP using CIRBP antibody (Invitrogen, Carlsbad, CA, USA). Quantification of immunohistochemical staining was scored based on the percentage of positive stained cells and the staining intensity. Staining in more cores was analyzed to standardize the analysis.

### 2.3. Cell Culture

Two pancreatic cancer cell lines, BxPC-3 and PANC-1, were purchased from ATCC (Manassas, VA, USA). BxPC-3 and PANC-1 cells were cultured in RPMI-1640 Medium (Invitrogen) and Dulbecco's Modified Eagle Medium (DMEM; Invitrogen), which was supplemented with 10% fetal bovine serum (Invitrogen) at 37 or 32°C in a humidified atmosphere of 5% CO_2_.

### 2.4. RNA-Binding Protein Immunoprecipitation (RIP) Assay

Pancreatic cancer cells were washed and then lysed with RIP lysis buffer on ice for 10 min. After that, the cell lysates were centrifuged at 14,000 × g and 4°C for 10 min, and the supernatants were collected. Magnetic Beads were precoated with 10 *μ*g of either anti-CIRBP or Normal Rabbit IgG (Millipore, Billerica, MA, USA) at 4°C overnight and then resuspended in RIP Immunoprecipitation Buffer. Next, the bead-antibody complexes were mixed with the supernatants at 4°C overnight. Total RNA was isolated, and the mRNA levels of p53 were analyzed using quantitative real-time PCR (qRT-PCR).

### 2.5. qRT-PCR

Total RNA was extracted from pancreatic cancer cells using Trizol (Invitrogen) and then reverse transcribed into cDNA using a PrimeScript II 1st Strand cDNA Synthesis Kit (TaKaRa, Beijing, China). qPCR was performed using the SYBR Premix Ex Taq II (Perfect Real Time; TaKaRa) in ABI PRISM 7500 Real-Time PCR System. The relative mRNA expression levels of CIRBP and p53 were calculated with 2^−*ΔΔ*CT^ method. The primers are as follows: CIRBP-F1—5′-TGGTGGTTGTGAAAGACAGG-3′, CIRBP-R1—5′-GCCGTCCATCTACAGACTTC-3′; P53-F1—5′-TTCTACAGTTGGGCAGCT-3′, P53-R1—5′-GCAGTAAGCCAAGATCAC-3′.

### 2.6. Western Blot Analysis

Pancreatic cancer cells were lysed in RIPA buffer (Beyotime, Shanghai, China) supplemented with a protease and phosphatase inhibitor cocktail (Beyotime) to extract protein. Equal amounts of protein were electrophoresed and then transferred onto PVDF membranes (Millipore). After that, blotted membranes were blocked with 5% skim milk and incubated with anti-CIRBP (1 : 1000; Abcam, Shanghai, China), anti-p53 (1 : 1000; Abcam), anti-dipeptidyl peptidase 4 (DPP4; 1 : 1000; Abcam), anti-glutathione peroxidase 4 (GPX4; 1 : 1000; Abcam), anti-NADPH oxidase 1 (NOX1; 1 : 500; Abcam), anti-ferritin heavy chain 1 (FTH1; 1 : 500; Abcam), or *β*-actin (1000; Cell Signaling Technology, Shanghai, China) primary antibody. Then, the membranes were incubated with an anti-rabbit IgG HRP-linked antibody (Cell Signaling Technology) for 1 h, and the bands were visualized using the BeyoECL Plus (Beyotime).

### 2.7. Prussian Blue Staining

The treated cells were fixed with 95% ethanol for more than 60 min, and intracellular iron was visualized using Prussian blue staining. The cells were incubated with Prussian blue staining solution (2% potassium ferrous hydride aqueous solution and 2% hydrochloric acid were mixed at an equal ratio) for 20 min and then washed with distilled water. After Eosin staining, the cells were placed under a light microscope for observation.

### 2.8. Measurement of Glutathione Peroxidase (GSH-Px) Activity and Mitochondrial Reactive Oxygen Species (ROS)

GSH-Px was measured using a GSH-Px assay kit (Nanjing Jiancheng Bioengineering Institute, Nanjing, China). Pancreatic cancer cells were incubated with MitoTracker™ Red FM (200 nmol/L; Invitrogen) for 30 min at 37°C, followed by an incubation with DCFH-DA (10 *μ*mol/L; Beyotime) for 20 min at 37°C. After that, the colocalization of ROS and mitochondria was analyzed by confocal imaging.

### 2.9. Cell Viability Assay

Briefly, 1 × 10^4^ pancreatic cancer cells in 100 *μ*L culture media were seeded in a 96-well plate, then added with ferroptosis inhibitor Ferrostatin-1 (Fer-1, 10 *μ*mol/L; MedChemExpress, Shanghai, China), and cultured at 32°C. Then, the cells were incubated with CCK-8 solution (10 *μ*L/well, Beyotime) for 4 h at 37°C. The optical density in each well was quantified at 450 nm wavelength with a microplate reader.

### 2.10. Cell Apoptosis

Pancreatic cancer cells were stained with 1.25 *μ*L Annexin V-FITC (keygen, China) for 15 min and then stained with 10 *μ*L propidium iodide for 30 min. The apoptosis rate was immediately measured by flow cytometry.

### 2.11. Statistical Analysis

The data are presented as the mean ± standard deviation (SD). Group comparisons of CIRBP expression were performed using a nonparametric test. A survival curve was prepared using the Kaplan-Meier method with the log-rank test. Statistical analysis was performed with Student's *t*-test, and a *P* value < 0.05 was considered significant.

## 3. Results

### 3.1. The Expression Level of CIRBP Is Closely Related to Pancreatic Cancer

**Figure 1:**
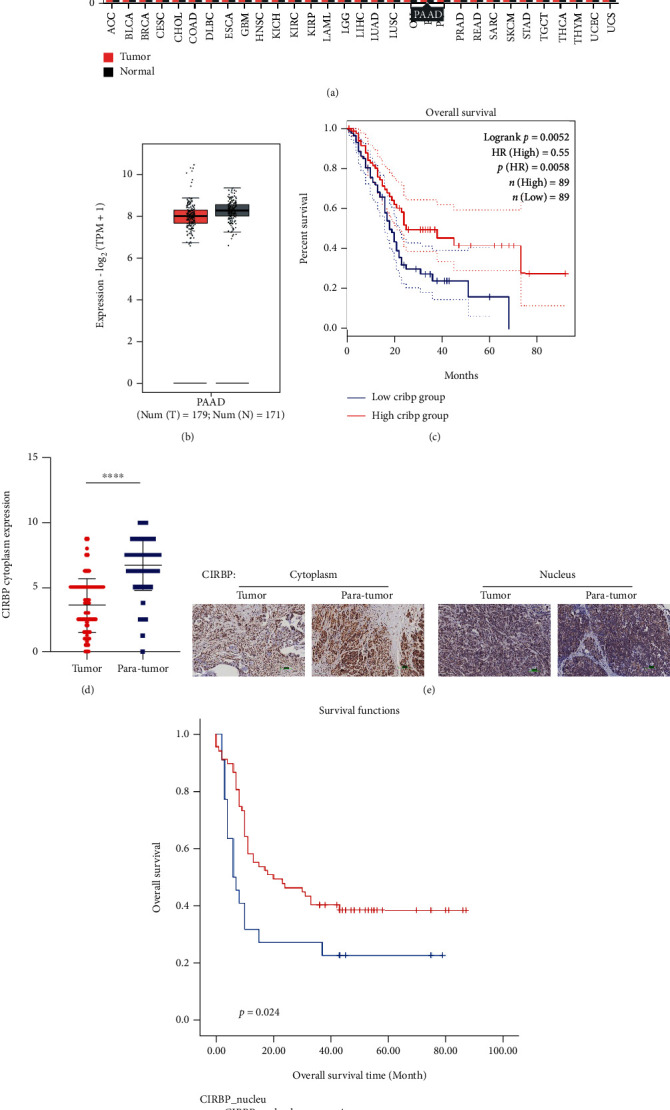
The expression level of CIRBP in pancreatic cancer. (a) The online database GEPIA2 showed the expression level of CIRBP in different tumors. (b) Data from database GEPIA2 showed the expression level of CIRBP in pancreatic cancer. (c) Survival curve analysis was performed using the expression data of CIRBP in database GEPIA2. (d) The expression level of CIRBP in the cytoplasm of cancer tissues was significantly lower than that of adjacent tissues. ^∗∗∗∗^*P* < 0.0001 versus tumor. (e) The expression level of CIRBP in pancreatic cancer tissues was detected by immunohistochemistry. (f) Survival curve was analyzed according to the expression level of CIRBP in pancreatic cancer tissues.

Use the online tool GEPIA2 (http://gepia2.cancer-pku.cn/#index) to check the expression level of CIRBP; it was found that the expression of CIRBP in pancreatic cancer tissues was slightly lower than that in normal tissues (the difference is not significant; Figures 1(a) and 1(b)), and the survival curve analysis showed that the high expression of CIRBP was beneficial to survival (the difference was statistically significant; Figure 1(c)). Previous studies have shown that different expression locations of CIRBP can affect its function [14]. Therefore, the research further used immunohistochemistry to detect the expression location of CIRBP in pancreatic cancer tissues. The results showed that the expression level of CIRBP in the cytoplasm of cancer tissues was significantly lower than that of adjacent tissues (Figures 1(d) and 1(e)). Although the expression level of CIRBP in the nucleus of cancer tissues was not significantly different from that of adjacent tissues (Figure 1(e)), survival curve analysis found that pancreatic cancer patients with high nuclear CIRBP expression had longer overall survival (Figure 1(f)). These results indicate that CIRBP may play a tumor suppressor effect in the progression of pancreatic cancer.

### 3.2. Cold Induction Regulates the Expression of Key Factors of Ferroptosis through CIRBP/p53 Pathway

**Figure 2:**
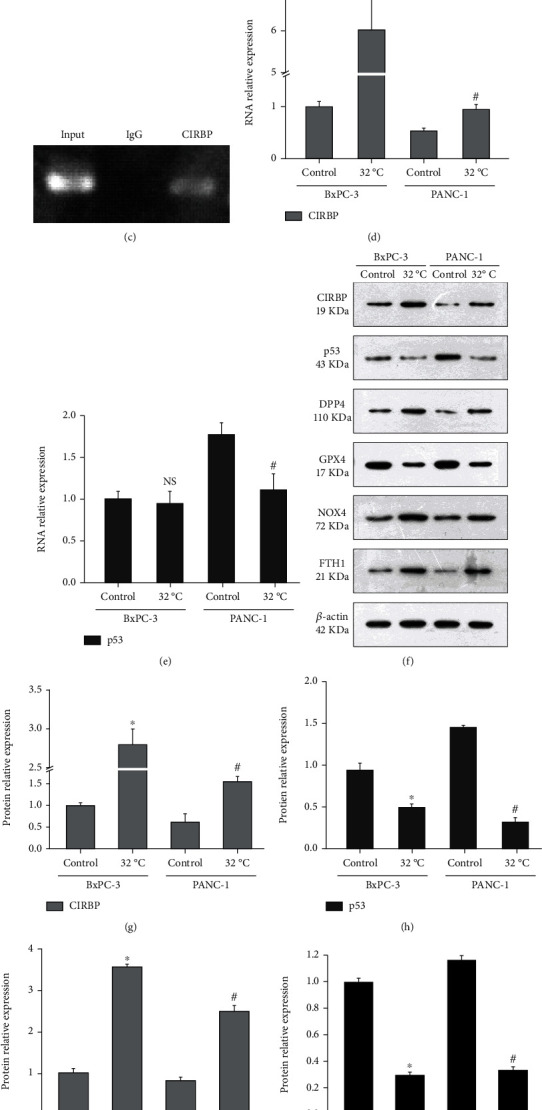
Cold induces CIRBP expression to affect the expression of key regulatory factors of ferroptosis. (a) Searching for factors that interact with CIRBP in the GeneCards database. (b) RIP showed that CIRBP antibody significantly enriched p53 RNA. ^∗^*P* < 0.05 versus IgG; ^#^*P* < 0.05 versus CIRBP. (c) Agarose gel electrophoresis showed the expression of p53. BxPC-3 and PANC-1 cells were cultured at 37 (Control) or 32°C, then the expression of CIRBP (d) and p53 (e) was detected by qRT-PCR, and western blot analysis (f) was used to detect the protein expression levels of CIRBP (g), p53 (h), DPP4 (i), GPX4 (j), NOX1 (k), and FTH1 (l). ^∗^*P* < 0.05 versus Control-BxPC-3; ^#^*P* < 0.05 versus Control-PANC-1.

Searching for factors that interact with CIRBP in the GeneCards (https://www.genecards.org/) database found that CIRBP was closely related to the p53/GPX4 pathway (Figure 2(a)). The online tool RNA-Protein Interaction Prediction (http://pridb.gdcb.iastate.edu/RPISeq/) analysis also showed that CIRBP could combine with the 3′-UTR of p53 gene. Furthermore, based on the prediction by catRAPID (http://service.tartaglialab.com/page/catrapid_group), primers were designed to detect the predicted binding region. The results of RIP found that CIRBP antibody indeed significantly enriched p53 RNA (Figures 2(b) and 2(c)), indicating that CIRBP can directly bind to p53. CIRBP is a cold-induced RNA-binding protein, so pancreatic cancer cells were treated with cold, and it was found that cold induction did significantly promote the expression of CIRBP (Figure 2(d)). Cold induction did not affect the expression of p53 mRNA in BxPC-3 cells but significantly inhibited its expression in PANC-1 cells (Figure 2(e)). Further detection of protein expression level showed that cold induction promoted the expression of CIRBP while inhibiting the expression of p53 and, at the same time, promoted the expression of DPP4 (Figures 2(f)–2(i)). In addition, cold induction significantly inhibited the expression of GPX4 but upregulated the expression of NOX1 and FTH1 (Figures 2(f) and 2(j)–2(l)). The above genes are the key factors regulating the progress of ferroptosis. Therefore, cold induction affects the process of ferroptosis by inducing the expression of CIRBP and then regulating key factors such as p53 and GPX4.

### 3.3. Cold-Induction Promotes the Occurrence of Ferroptosis in Pancreatic Cancer Cells

**Figure 3:**
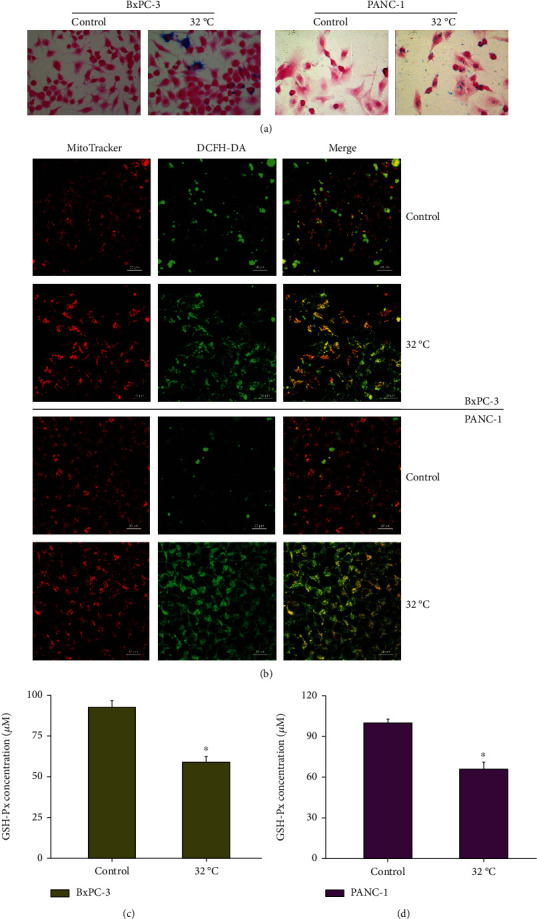
Cold induces Fe^2+^ accumulation and abnormal oxidative stress. BxPC-3 and PANC-1 cells were cultured at 37 (Control) or 32°C. (a) The increase in Prussian blue staining indicated an increase in Fe^2+^ accumulation level. (b) Representative confocal fluorescent images of MitoTracker™ Red FM (the mitochondrial indicator) and DCFH-DA (ROS indicator)-loaded cells. (c) GSH-Px activity was measured using a GSH-Px assay kit. ^∗^*P* < 0.05 versus Control.

Further detection of ferroptosis key indicators showed that cold induction enhanced the accumulation of Fe^2+^ in cells and promoted the production of ROS (Figures 3(a) and 3(b)). In addition, cold induction inhibited the expression of GSH-Px (Figure 3(c)). Similar results were found in another pancreatic cancer cell line PANC-1 (Figure 3). The increase in Prussian blue staining indicated an increase in Fe^2+^ accumulation level (Figure 3(a)); the fluorescence of DCFH-DA was significantly increased; that is, the generation of ROS was significantly increased (Figure 3(b)); at the same time, the concentration of GSH-Px was decreased (Figure 3(d)). Therefore, cold induction significantly enhances the accumulation of Fe^2+^ and ROS, thereby promoting the occurrence of ferroptosis.

### 3.4. Cold Induces Ferroptosis to Inhibit Cell Proliferation and Promote Apoptosis

**Figure 4:**
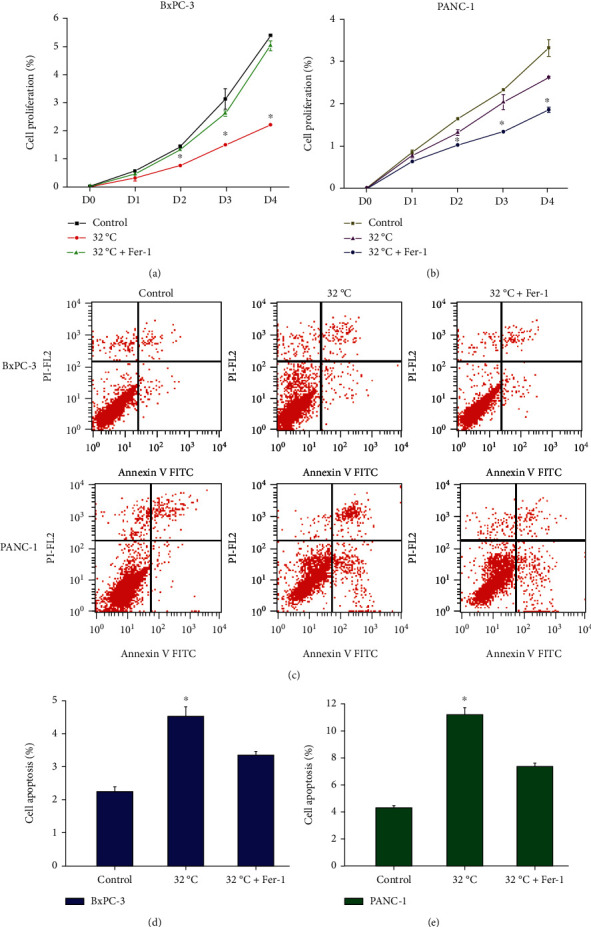
Cold induces ferroptosis to regulate the proliferation and apoptosis of pancreatic cancer cells. BxPC-3 and PANC-1 cells were cultured at 37°C (Control), 32°C, or treated with ferroptosis inhibitor Ferrostatin-1 (Fer-1) and then cultured at 32°C (32°C+Fer-1). The proliferation of BxPC-3 (a) and PANC-1 (b) cells was detected by CCK-8 assay. (c–e) Detection of apoptosis by flow cytometry. ^∗^*P* < 0.05 versus Control.

Cold induction significantly inhibited the proliferation of pancreatic cancer cells, but with the addition of ferroptosis inhibitor Fer-1, cell proliferation was significantly restored, and similar results were found in different cell lines (Figures 4(a) and 4(b)). This indicates that cold induction can activate ferroptosis to inhibit the proliferation of pancreatic cancer cells. At the same time, the detection of apoptosis found that cold induction significantly enhanced the apoptosis of pancreatic cancer cells, but the ferroptosis inhibitor Fer-1 blocked the effect of apoptosis induction by cold (Figures 4(c)–4(e)). This indicates that cold-induced ferroptosis is also related to the progression of apoptosis.

## 4. Discussion

Despite the continuous emergence of new advances in the field of comprehensive treatment of pancreatic cancer, it still has little effect in improving the prognosis of patients, and the 5-year survival rate is still less than 5% [2]. Therefore, in order to improve the early diagnosis, treatment, and prognosis of patients, exploring the molecular mechanism of pancreatic cancer pathogenesis, and finding molecular markers and new therapeutic targets for early diagnosis and prognosis has become an urgent problem to be solved.

Previous studies have shown that CIRBP is closely related to the occurrence and development of tumors and plays an important role in a variety of tumors [5]. This study found that the expression of CIRBP in pancreatic cancer tissues was lower than that in adjacent normal tissues and the high expression of CIRBP was beneficial to survival, indicating that CIRBP exerts a tumor suppressor function in pancreatic cancer. Jang et al. [7] also showed that the expression level of CIRBP was closely related to the prognosis of colon cancer patients, and the 5-year survival rate of patients with high expression of CIRBP was better than that of patients with low expression of CIRBP, suggesting that CIRBP may play a role of tumor suppressor gene in colon cancer. Compared with normal endometrial cells, CIRBP expression was low in endometrial cancer cells, and the expression level of CIRBP was negatively correlated with the cell proliferation marker Ki-67, which suggests that inhibition of CIRBP expression may be related to the formation of endometrial cancer [15]. A large sample of ovarian cancer microarray study found that the expression of CIRBP was downregulated in ovarian cancer; further, in vitro cell experiments showed that the upregulation of CIRBP significantly inhibited the proliferation of ovarian cancer cells [16]. These studies indicate that CIRBP does have a tumor suppressor effect in some tumors such as pancreatic cancer.

As an RNA-binding protein, CIRBP can regulate target gene expression by specifically binding to the 3′-untranslated regions (3′-UTR) of target gene mRNA [17]. This study showed that CIRBP could directly bind to p53. Lee et al. [12] found that overexpression of CIRBP downregulated the expression level of p53. Therefore, the role of CIRBP in pancreatic cancer is closely related to p53. Online database analysis combined with cell experiments further found that cold treatment of pancreatic cancer cells significantly induced the expression of CIRBP, DPP4, NOX1, and FTH1, while inhibiting the expression of p53 and GPX4. Xie et al. [18] found that p53 deletion in colorectal cancer inhibited the nuclear accumulation of DPP4, which in turn promoted DPP4-dependent lipid peroxidation and then induced ferroptosis. NOX1, FTH1, and GPX4 are the key regulators of ferroptosis [19]. Therefore, cold-induced CIRBP expression can activate the downstream ferroptosis pathway through p53. This study further found that cold induction enhanced Fe^2+^ accumulation in cells, promoted the generation of ROS, and inhibited the expression of GSH-Px. This further confirmed that cold induction could promote the process of ferroptosis by inducing the expression of CIRBP and then regulating key factors such as p53 and GPX4. Ferroptosis inducer erastin or hypoxia-reoxygenation (HR) treatment induced renal tubular epithelial cells to express CIRBP, and silencing of CIRBP inhibited ferroptosis induced by HR or erastin [20]. Therefore, CIRBP is also a key factor regulating the progression of ferroptosis.

Cold induction significantly inhibited proliferation and induced apoptosis of pancreatic cancer cells, but after the addition of ferroptosis inhibitor, cell proliferation and apoptosis did not change significantly. This indicates that cold induces CIRBP expression and then activates ferroptosis, thus inhibiting the growth of pancreatic cancer cells. Yamaguchi et al. [21] showed that piperlongumine depleted GSH and led to an increase in intracellular lipid ROS, which led to ferroptosis of pancreatic cancer cells. Ou et al. [22] found that SAT1, a target gene downstream of p53, caused lipid peroxidation and ferroptosis by upregulating the expression of ALOX15. Shintoku et al. [23] showed by immunofluorescence analysis that in PANC-1 pancreatic cancer cells, ALOX15 was localized on cell membrane, which could oxidize polyunsaturated fatty acids (PUFA) and then increased the sensitivity of cells to ferroptosis inducer RSL3. Therefore, induction of ferroptosis can cause the death of pancreatic cancer cells, and at the same time, it can play a synergistic effect with other anticancer drugs to improve the sensitivity of pancreatic cancer to existing therapies, indicating that induction of ferroptosis for pancreatic cancer may have great potential.

In conclusion, CIRBP is downregulated in pancreatic cancer and can induce ferroptosis by regulating the p53/GPX4 pathway, thereby inhibiting cell growth and playing the role of tumor suppressor gene, indicating that it may be an important target for the diagnosis and treatment of pancreatic cancer.

## Data Availability

The data used to support the findings of this study are included within the article.
